# Diagnostic performance of diffusion-weighted imaging versus 18F-FDG PET/CT in differentiating pulmonary lesions: an updated meta-analysis of comparative studies

**DOI:** 10.1186/s12880-023-00990-y

**Published:** 2023-03-10

**Authors:** Jieqiong Liu, Xiaoying Xia, Qiao Zou, Xiaobin Xie, Yongxia Lei, Qi Wan, Xinchun Li

**Affiliations:** Department of Radiology, The First Affiliated Hospital of Guangzhou Medical University, Yanjiangxilu No 151, Guangzhou, 510120 China

**Keywords:** Magnetic resonance imaging, Positron emission tomography/computed tomography, Lung neoplasms, Diffusion-weighted imaging, Pulmonary nodule

## Abstract

**Objective:**

To compare the diagnostic accuracy of diffusion-weighted imaging (DWI) and 18F-fluorodeoxyglucose positron emission tomography/computed tomography (18F-FDG PET/CT) for differentiating pulmonary nodules and masses.

**Methods:**

We systematically searched six databases, including PubMed, EMBASE, the Cochrane Library, and three Chinese databases, to identify studies that used both DWI and PET/CT to differentiate pulmonary nodules. The diagnostic performance of DWI and PET/CT was compared and pooled sensitivity and specificity were calculated along with 95% confidence intervals (CIs). The Quality Assessment of Diagnostic Accuracy Studies 2 was used to assess the quality of the included studies, and STATA 16.0 software was utilized to perform statistical analysis.

**Results:**

Overall, 10 studies that enrolled a total of 871 patients with 948 pulmonary nodules were included in this meta-analysis. DWI had greater pooled sensitivity (0.85 [95% CI 0.77–0.90]) and specificity (0.91 [95% CI 0.82–0.96]) than PET/CT (sensitivity, 0.82 [95% CI 0.70–0.90]); specificity, (0.81, [95% CI 0.72–0.87]). The area under the curve of DWI and PET/CT were 0.94 (95% CI 0.91–0.96) and 0.87 (95% CI 0.84–0.90) (Z = 1.58, *P* > 0.05), respectively. The diagnostic odds ratio of DWI (54.46, [95% CI 17.98–164.99]) was superior to that of PET/CT (15.77, [95% CI 8.19–30.37]). The Deeks’ funnel plot asymmetry test showed no publication bias. The Spearman correlation coefficient test revealed no significant threshold effect. Lesion diameter and reference standard could be potential causes for the heterogeneity of both DWI and PET/CT studies, and quantitative or semi-quantitative parameters used would be a potential source of bias for PET/CT studies.

**Conclusion:**

As a radiation-free technique, DWI may have similar performance compare with PET/CT in differentiating malignant pulmonary nodules or masses from benign ones.

**Supplementary Information:**

The online version contains supplementary material available at 10.1186/s12880-023-00990-y.

## Introduction

Due to the high morbidity and mortality rates of lung cancer worldwide [1], differentiating malignant from benign pulmonary nodules is critical. The early detection of lung cancer could improve the survival rate and reduce mortality rate and a complete treatment could be achieved by early tumor resection. In clinical practice, computed tomography (CT) has been widely used for discriminating malignant nodules from benign pulmonary nodules. However, due to the overlap of morphological signs, distinguishing malignant nodules from benign nodules only based on CT findings is a clinical challenge for radiologists and physicians.

Positron emission tomography/computed tomography (PET/CT), as a widely used technique in clinical practice, provides combined metabolic and morphological information, which has made a great contribution to staging lung cancer, detecting metastatic lymph nodes, and evaluating the response to treatment in lung cancer patients [2–6]. In addition, PET/CT has been used for differentiating the malignant nodules from benign pulmonary nodules, and it has shown a higher accuracy than CT [5–7].

Recently, with the rapid development of magnetic resonance imaging (MRI) techniques, diffusion-weighted imaging (DWI) has gradually become an alternative for diagnosing pulmonary lesions. It can quantitatively provide an apparent diffusion coefficient (ADC), which reflects the cellularity of biological tissues [8]. According to previous studies, DWI has shown to be advantageous not only for predicting lung cancer invasiveness and pathological type of pulmonary tumors, but also for discriminating malignant nodules from benign pulmonary nodules [9–11].

To date, there is only one meta-analysis that compared the diagnostic performance of DWI and PET/CT in differentiating malignant and benign nodules/masses [12]. However, it included only literature in English and failed to conduct a subgroup analysis in their primary analysis because of the small amount of included studies [12]. Dividing the studies into subgroups based on specific characteristics (e.g. lesion diameter, imaging modality, study design) is important and can help identify the sources of heterogeneity of published papers. Moreover, there have been a few relevant comparative studies published in English and Chinese during the past several years. Therefore, we aimed to perform an updated meta-analysis of comparative studies to conduct a comprehensive and detailed analysis of this topic and conducted subgroup analyses to further explore the influencing factors of DWI and PET/CT in differentiating pulmonary nodules.

## Materials and methods

### Search strategy

The PubMed, EMBASE, the Cochrane Library, Wanfang, China National Knowledge Infrastructure (CNKI), and VIP databases were systematically searched from inception until June 2021 to retrieve comparative studies related to the diagnostic performance of DWI and PET/CT. Two radiologists assessed titles and abstracts separately for identification of potentially eligible studies. The keywords that were used to comprehensively search for the relevant articles in the above-mentioned databases are presented in Additional file 1.

### Inclusion and exclusion criteria

Reviewers selected relevant studies that met the following inclusion criteria: (1) patients with pulmonary nodules who underwent both DWI and PET/CT in the same period; (2) the data of true-positive (TP), true-negative (TN), false-positive (FP), and false-negative (FN) were clearly reported; (3) the characteristics of pulmonary nodules should be detected by histological examination after surgical resection or imaging follow-up of more than 2 years without changing.

### Data extraction and quality assessment

Two radiologists independently extracted the main characteristics of all the included articles, as shown in Tables 1 and 2, to reduce potential bias, and blindly evaluated the risk of bias using the Quality Assessment of Diagnostic Accuracy Studies 2 (QUADAS-2) [13]. All disagreements were resolved through consensus.

**Table 1:** main characteristics of 10 studies included in the meta-analysis

References		Inclusion interval	Country	Mean age (range)	Design	No. of patients (F/M)	No. of nodules	Magnetic strength	b-values (s/mm^2^)	Parameter (DWI, PET)	Reference test
Ohno et al. [18]		2015.09–2016.08	Japan	70.3 (53–84)	Prospective	82 (30/52)	88	3.0T	1000	ADC, SUV_max_	His/Fol
Nomori et al. [19]		2012.12–2014.03	Japan	–	Retrospective	77 (–/–)	87	1.5T	800	SI-CR, SUV-CR	His/Fol
Ohba et al. [24]		2007.10–2009.03	Japan	–	Prospective	58 (–/–)	76	3.0T, 1.5T	1000	ADC, SUV-CR	His/Fol
Mori et al. [25]		2006.02–2007.07	Japan	68 (20–80)	Retrospective	104 (49/55)	140	1.5T	1000	ADC, SUV-CR	His/Fol
Ohba et al. [8]		2006.02–2007.12	Japan	68 (36–82)	Retrospective	110 (54/56)	124	1.5T	1000	ADC, SUV-CR	His/Fol
Zhang et al. [26]		2010.09–2013.01	Chineses	59 (26–81)	Retrospective	113 (46/67)	108	3.0T	1000	ADC, SUV_max_	His
Katsuo et al. [22]		2009.05–2012.02	Japan	68.5 (37–87)	Prospective	189 (65/124)	189	1.5T	800	ADC, SUV_max_	His/Fol
Kang et al. [21]		2008.09–2009.06	Chineses	51 (39–78)	Retrospective	26 (5/21)	26	1.5T	600	ADC, SUV_max_	His/Fol
Liu et al. [23]		2016.01–2018.06	Chineses	64.6 (–)	Prospective	36 (12/24)	36	3.0T	Total	ADC, SUV_max_	His
Selcuk et al. [20]		2014–2017	Turkey	57.8 (36–85)	Prospective	76 (16/60)	74	1.5T	500	ADC, SUV_max_	His

*His* Histological, *Fol* Follow- up

**Table 2:** Pooled analyses of DWI and PET/CT for the evaluation of pulmonary lesions in including studies respectively

References		DWI						PET/CT				
		Magnetic strength	Parameter (cut off value)	TP	FP	FN	TN	Parameter (cut off value)	TP	FP	FN	TN
Ohno et al. [18]		3.0T	ADC (1.23 × 10^−3^ mm^2^/s)	33	14	16	25	SUV_max_ (2.78)	24	3	25	36
Nomori et al. [19]		1.5T	SI-CR (0.45)	57	2	9	19	SUV-CR (2.65)	47	4	19	17
Ohba et al. [24]		3.0T	ADC (1.85 × 10^−3^ mm^2^/s)	52	1	6	17	SUV-CR (1.7)	51	1	7	17
		1.5T	ADC (1.1 × 10^−3^ mm^2^/s)	53	1	5	17					
Mori et al. [25]		1.5T	ADC (1.1 × 10^−3^ mm^2^/s)	74	1	32	33	SUV-CR (0.37)	76	7	30	27
Ohba et al. [8]		1.5T	ADC (1.2 × 10^−3^ mm^2^/s)	70	1	26	27	SUV-CR (0.31)	69	5	27	23
Zhang et al. [26]		3.0T	ADC (0.99 × 10^−3^ mm^2^/s)	72	3	5	33	SUV_max_ (6.5)	64	12	13	24
Katsuo et al. [22]		1.5T	ADC (1.44 × 10^−3^ mm^2^/s)	128	10	32	19	SUV_max_ (3.43)	112	10	48	19
Kang et al. [21]		1.5T	ADC (-)	18	2	1	5	SUV_max_ (-)	19	3	0	4
Liu et al. [23]		3.0T	ADC (1.05 × 10^−3^ mm^2^/s)	17	0	9	10	SUV_max_ (5.5)	25	3	1	7
Selcuk et al. [20]		1.5T	ADC (1.14 × 10^−3^ mm^2^/s)	39	1	1	35	SUV_max_ (3.75)	38	3	2	33

*TP* True-positive, *FP* False-positive, *FN* False-negative, *TN* True-negative, *ADC* Apparent diffusion coefficient, *SUVmax* The maximum standard uptake value, *SI-CR* Apparent diffusion coefficient and the contrast ratio between the lesions and spinal cord of signal intensity, *SUV-CR* The maximum standard uptake value and the contrast ratio between the lesions and contralateral lung of standard uptake value

### Statistical analysis

A bivariate random-effects model was used to not only calculate the pooled sensitivities and specificities with 95% confidence intervals (CIs), but also to estimate the pooled positive likelihood ratio (PLR), negative likelihood ratio (NLR), and diagnostic odds ratios (DORs) with 95% CIs [14]. Forest plots were used to assess variability via examining the study results visually. Moreover, I^2^ > 50% indicated as high heterogeneity, and a two-tailed *P*-value < 0.05 was considered statistically significant. To identify the sources of heterogeneity, subgroup analyses of DWI and PET/CT were separately carried out (Tables 3 and 4). Moreover, summary receiver operating characteristic (SROC) curves were plotted based on sensitivity (Y-axis) and specificity (X-axis) to indicate the diagnostic accuracy of PET/CT and DWI. To assess the effect of threshold, the Spearman correlation coefficient was used, in which was calculated between the logit of sensitivity and the logit of (1-specificity), and a correlation coefficient (ρ) > 0.6 was considered as a significant correlation. Finally, publication bias was assessed by the Deeks’ funnel plot asymmetry test [15]. All statistical analyses were conducted by STATA 16.0 (StataCorp LLC, College Station, TX, USA) and Meta-DiSc.

**Table 3:** Subgroup analysis for the diagnostic performance of DWI in detecting pulmonary lesions

Characteristics	Studies (n)	Pooled sensitivity (95% Cl)	Pooled specificity (95% Cl)
Total	11*	0.85 (0.77–0.90)	0.91 (0.82–0.96)
Year			
> 2011	6	0.85 (0.76–0.94)	**0.87 (0.76–0.97)**
≤ 2011	5	0.84 (0.74–0.94)	**0.95 (0.89–1.00)**
Design			
Prospective	6	0.85 (0.76–0.94)	0.89 (0.78–0.99)
Retrospective	5	0.84 (0.74–0.94)	0.93 (0.86–0.99)
No. of nodules			
≤ 100	7	0.87 (0.80–0.94)	0.91 (0.83–0.99)
> 100	4	0.81 (0.69–0.92)	0.91 (0.82–1.00)
Magnetic strength			
3T	4	0.83 (0.71–0.94)	0.89 (0.78–1.00)
1.5T	7	0.86 (0.78–0.94)	0.92 (0.85–0.99)
Lesion diameter**			
Only Nodules(≤ 3 cm)	4	**0.74 (0.64–0.85)**	0.91 (0.80–1.00)
Nodules and Masses	3	**0.87 (0.81–0.93)**	0.93 (0.85–1.00)
Disease in the benign group			
Only inflammation	3	0.90 (0.82–0.98)	0.94 (0.86–1.00)
Inflammation and tumors	8	0.82 (0.74–0.90)	0.90 (0.82–0.98)
Parameter			
ADC	10	0.84 (0.77–0.91)	0.91 (0.84–0.98)
SI-CR	1	0.87 (0.68–1.00)	0.92 (0.73–1.00)
Reference standard			
His/Fol	8	**0.82 (0.75–0.90)**	0.88 (0.79–0.97)
His	3	**0.90 (0.81–0.99)**	0.96 (0.91–1.00)

Boldface type indicates *P* value is < 0.05 in subgroup analysis of sensitivity or specificity; *His* Histological, *Fol* Follow-up

*A total of 10 studies were included but one study was counted twice due to use of both 1.5-T and 3.0-T MRI to conduct DWI on all patients, resulting in a total of 11 studies in the table

**Four studies were excluded from this subgroup due to lack of information on lesion diameter

**Table 4:** Subgroup analysis for the diagnostic performance of PET/CT in detecting pulmonary lesions

Characteristics	Studies (n)	Pooled sensitivity (95% Cl)	Pooled specificity (95% Cl)
Total	10	0.82 (0.70–0.90)	0.81 (0.72–0.87)
Year			
> 2011	6	0.86 (0.75–0.97)	0.81 (0.71–0.90)
≤ 2011	4	0.76 (0.59–0.93)	0.80 (0.69–0.92)
Design			
Retrospective	5	0.74 (0.58–0.90)	0.75 (0.65–0.84)
Prospective	5	0.90 (0.81–0.99)	0.87 (0.79–0.95)
No. of nodules			
≤ 100	6	0.81 (0.69–0.94)	0.84 (0.77–0.92)
> 100	4	0.83 (0.69–0.98)	0.74 (0.61–0.86)
Lesion diameter*			
Only nodules(≤ 3 cm)	4	**0.67 (0.58–0.76)**	0.86 (0.80–0.92)
Nodules and masses	2	**0.97 (0.93–1.00)**	0.82 (0.69–0.95)
Disease in the benign group			
Inflammation only	2	0.81 (0.60–1.00)	0.88 (0.76–1.00)
Inflammation and tumors	8	0.82 (0.71–0.93)	0.79 (0.70–0.87)
Parameter			
SUV_max_	6	0.86 (0.75–0.97)	**0.78 (0.68–0.88)**
SUV-CR	4	0.77 (0.60–0.93)	**0.84 (0.74–0.94)**
Reference standard			
His/Fol	7	**0.75 (0.65–0.85)**	0.82 (0.73–0.90)
His	3	**0.92 (0.84–0.99)**	0.79 (0.65–0.93)

Boldface type indicates *P* value is < 0.05 in subgroup analysis of sensitivity or specificity; *His* Histological, *Fol* Follow-up

*Four studies were excluded from this subgroup due to lack of information on lesion diameter

(http://www.hrc.es/investigacion/metadisc_en.htm) software.

### Analysis of diagnostic performance

The diagnostic performance of DWI and PET/CT was compared using the included studies. The parameters of both techniques used in the included studies were different, in which ADC and the contrast ratio between the lesions and spinal cord of signal intensity (SI-CR) were used in DWI, while the maximum standard uptake value (SUV_max_) and the contrast ratio between the lesions and contralateral lung of SUV (SUV-CR) were utilized in PET/CT.

## Results

### Selection of eligible studies and quality assessment

**Fig. 1:**
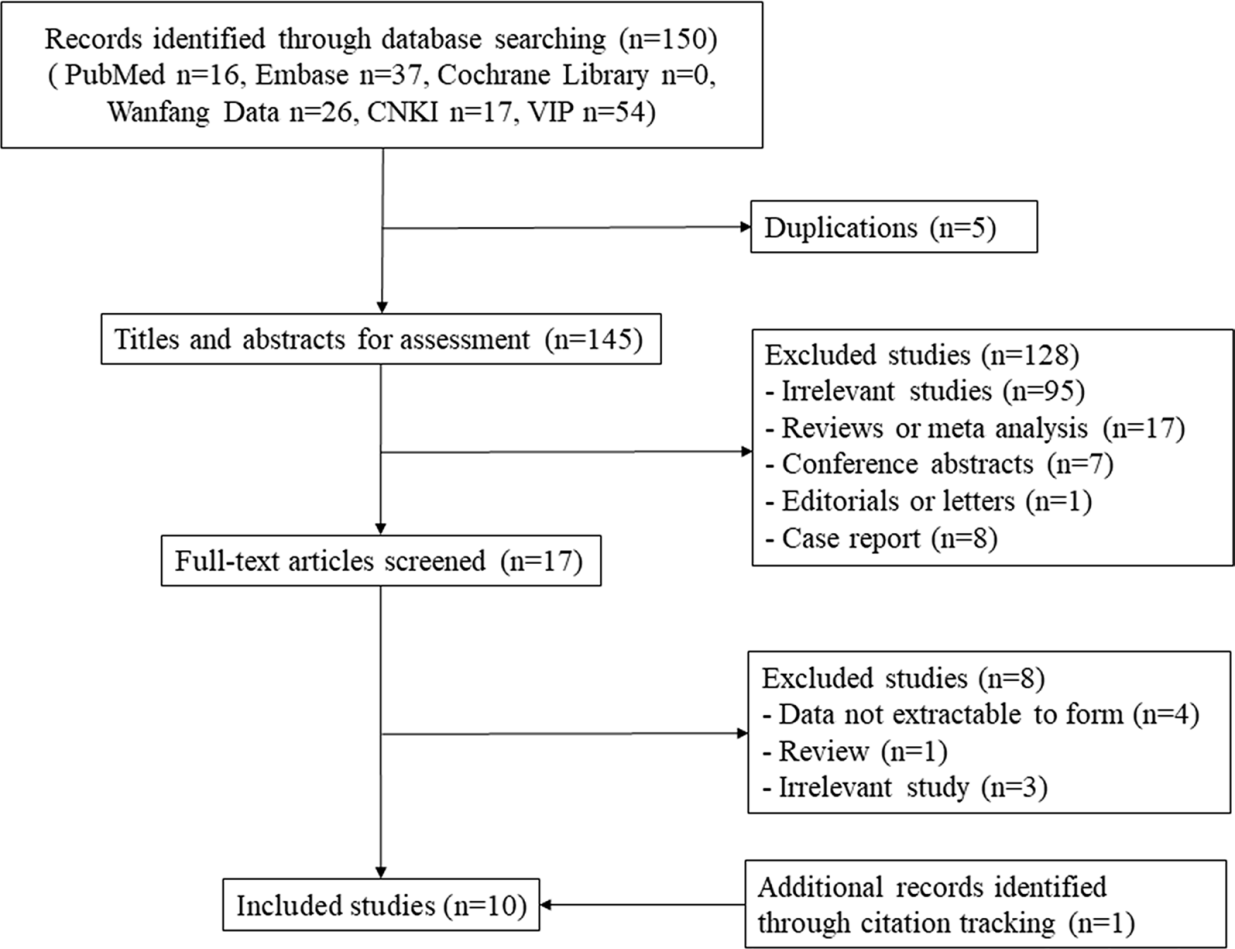
Flow chart for studies selection in the meta-analysis

After comprehensively searching in the online databases, 150 articles were retrieved, while most of studies were excluded after scanning the abstracts and titles by two reviewers independently. Finally, 10 articles that enrolled 871 patients with 948 pulmonary nodules who underwent both PET/CT and DWI in the same period were included (Fig. 1).

**Fig. 2:**
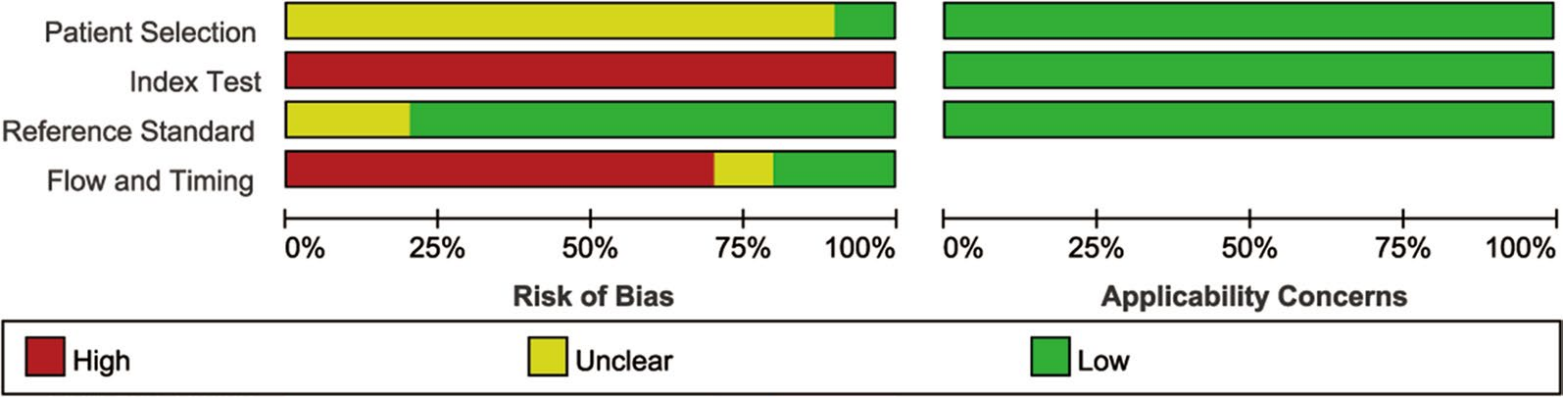
QUADAS-2 quality assessment of included studies

Methodological quality assessment was conducted among all the 10 studies with the QUADAS-2 tool (Fig. 2). Most of the studies were assessed as high risk in the index test domain and in the reference standard domain due to the use of an un-prespecified threshold or the different ways patients used as reference standard. As for the reference standard, samples that underwent histological diagnosis or follow-up of more than 2 years were identified [16, 17]. Therefore, only 2 studies were found with an unclear risk because they did not provide the appropriate time for radiological follow-up. What’s more, most of studies were also considered as unclear risk in the patients’ selection domain because the method (consecutive or random) of patients’ enrollment was not reported.

### Characteristics of the eligible studies

All characteristics of the included studies that were published from 2008 to 2020 are summarized in Table 1 [8, 18–26]. Overall, a total of 10 studies were included, 5 studies were prospective and the other 5 were retrospective. As for DWI, 3 studies used 3.0-T MRI machines, and 6 studies utilized 1.5-T MRI machines to perform the scanning, the other one study [24] used both 1.5-T and 3.0-T MRI to carry out the DWI scanning, thus being counted twice as we extracted both results to analyze separately.

Furthermore, most of the studies used ADC as the parameter, while only one study utilized SI-CR. As for PET/CT, on the other hand, SUV_max_ was used as an index of FDG uptake in 6 studies, and SUV-CR was considered in the other studies. Besides, 3 studies used histopathological findings only as the reference standard, while 7 studies combined follow-up data with histopathological findings to identify the biological behaviors of pulmonary lesions.

### Analysis of diagnostic accuracy and heterogeneity

**Fig. 3:**
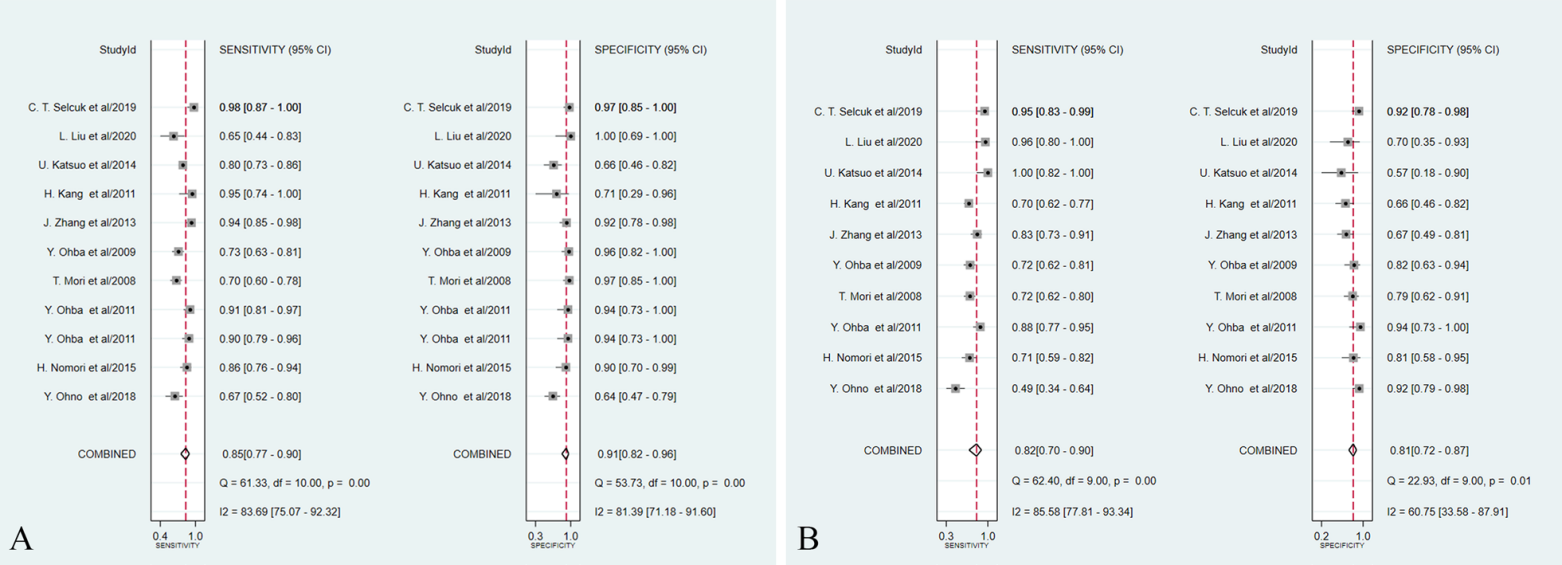
Forest plots of pooled sensitivity and specificity for **A** DWI and **B** PET/CT of 10 included studies. One DWI study [24] was counted twice due to the use of both 1.5-T and 3.0-T MRI on all patients, resulting in a total of 11 studies in the table. *DWI* Diffusion weighted imaging, *PET/CT* Positron emission tomography/computed tomography, *CI* Confidence interval

**Fig. 4:**
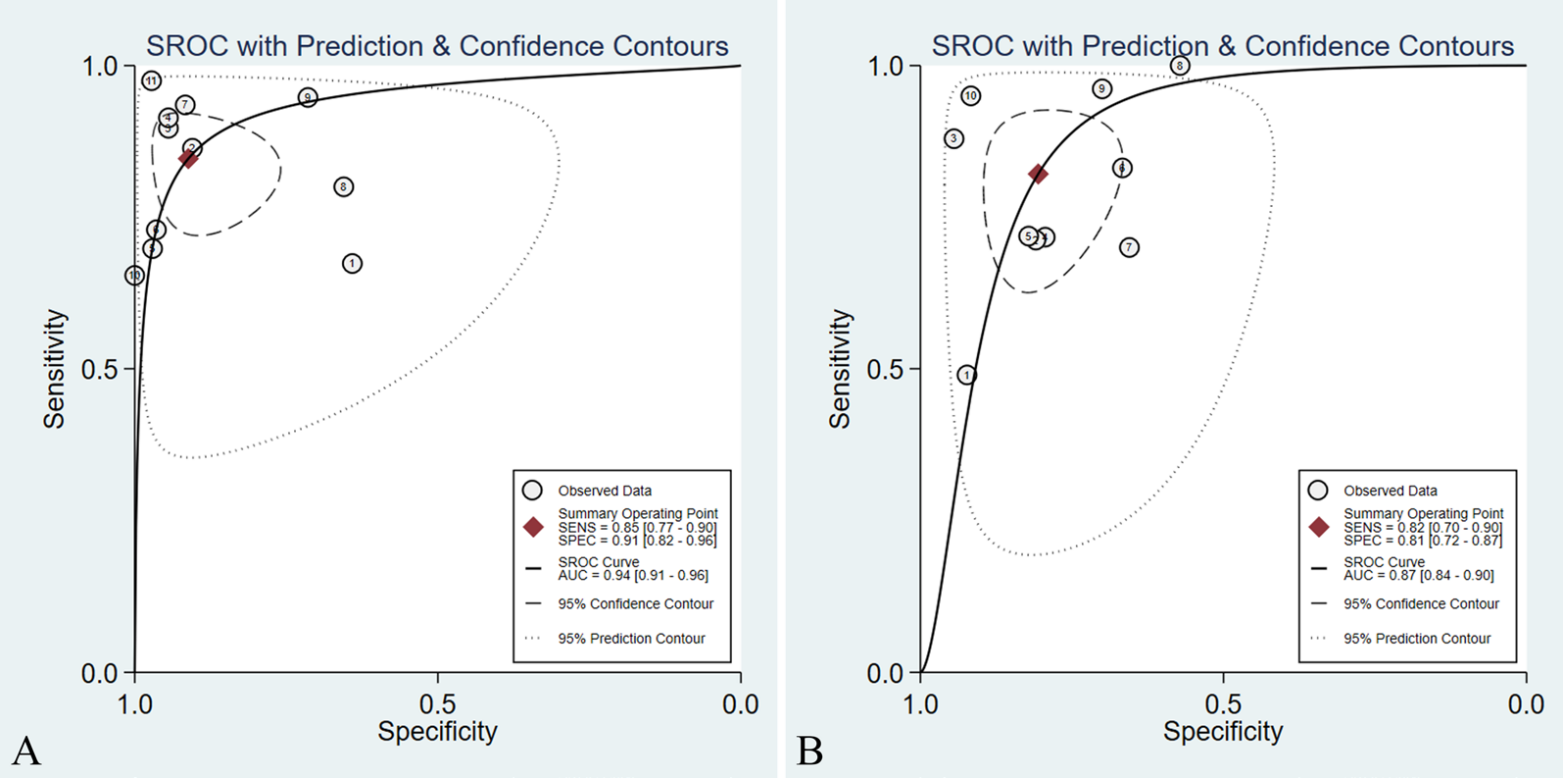
Summary receiver operating characteristics curves for **A** DWI and **B** PET/CT of 10 eligible studies. One DWI study [24] was counted twice due to the use of both 1.5-T and 3.0-T MRI on all patients, resulting in a total of 11 studies in the table. *AUC* Area under the curve, *SENS* Sensitivity, *SPEC* Specificity, *SROC* Summary receiver operating characteristics

**Fig. 5:**
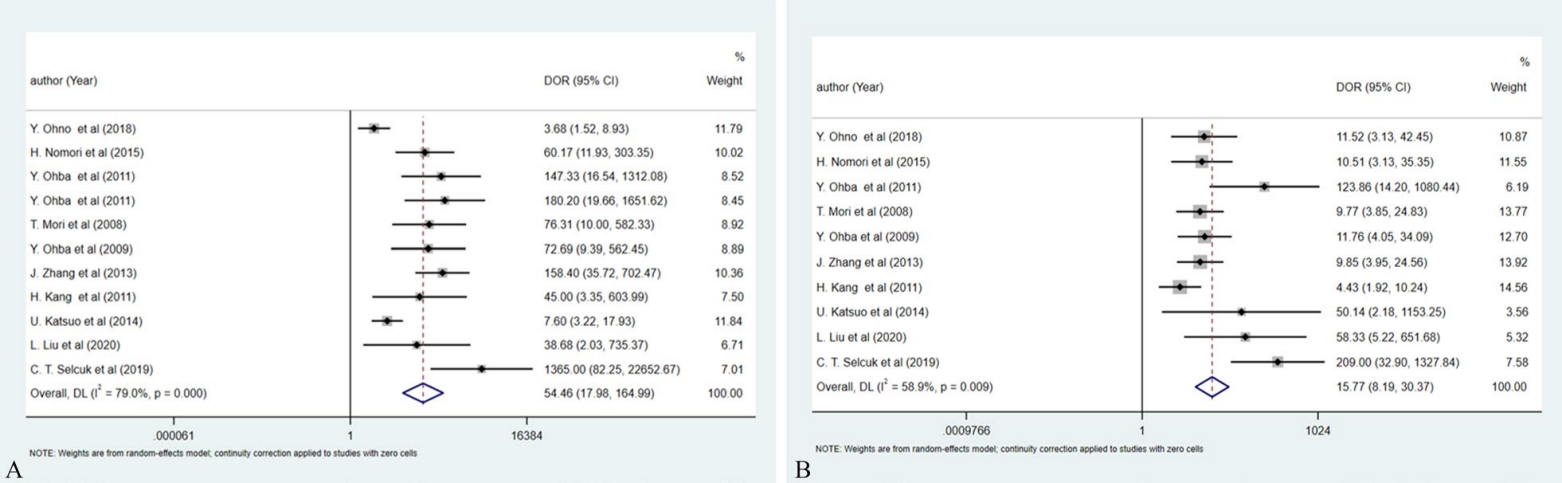
Forest plots of the estimated pooled diagnostic odds ratio **A** DWI and **B** PET/CT of 10 included studies. One DWI study [24] was counted twice due to the use of both 1.5-T and 3.0-T MRI on all patients, resulting in a total of 11 studies in the table. *DWI* Diffusion weighted imaging, *PET/CT* Positron emission tomography/computed tomography, *CI* Confidence interval

**Fig. 6:**
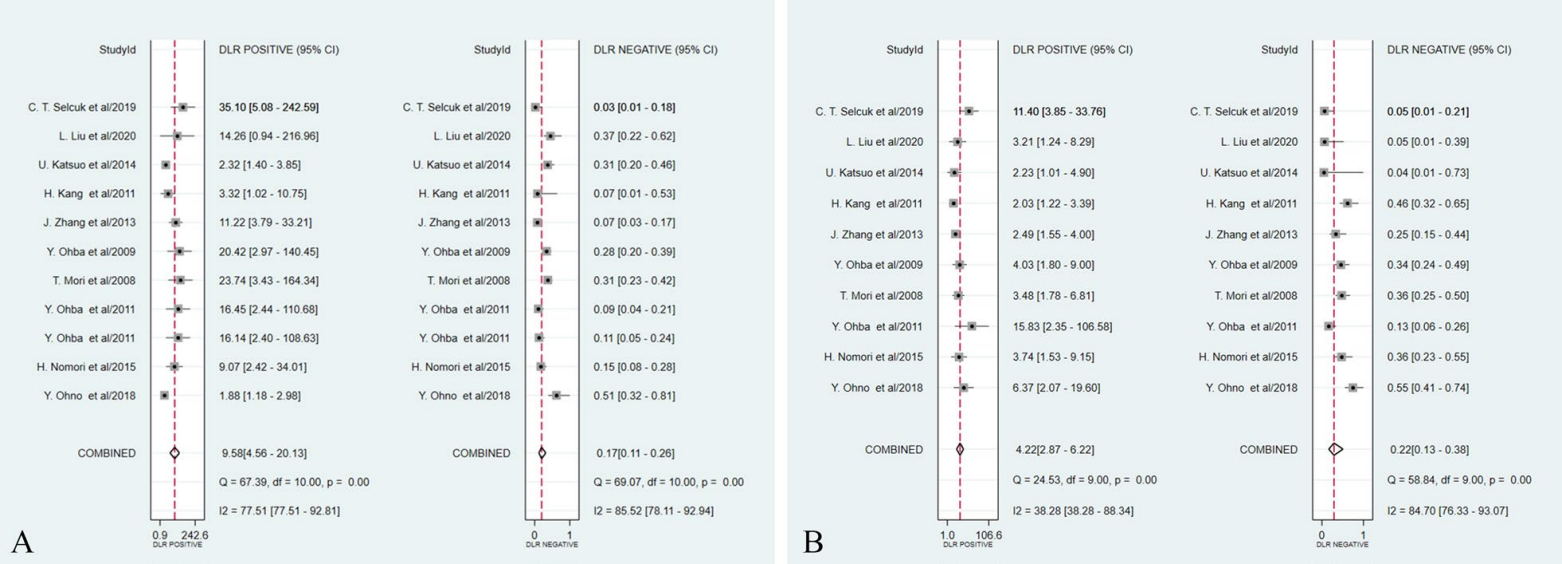
Forest plots of pooled diagnostic likelihood ratio **A** DWI and **B** PET/CT of 10 included studies. One DWI study [24] was counted twice due to the use of both 1.5-T and 3.0-T MRI on all patients, resulting in a total of 11 studies in the table. *DWI* Diffusion weighted imaging, *PET/CT* Positron emission tomography/computed tomography, *CI* Confidence interval

The results of the pooled analyses of the studies are presented in Table 1. As for DWI, the pooled sensitivity and specificity were 0.85 (95% CI 0.77–0.90) and 0.91 (95% CI 0.82–0.96), while the pooled sensitivity and specificity of PET/CT were 0.82 (95% CI 0.70–0.90) and 0.81 (95% CI 0.72–0.87), respectively (Fig. 3). Additionally, the area under the ROC curve (AUC) of DWI was 0.94 (95% CI 0.91–0.96), which was higher than that of 0.87 (95% CI 0.84–0.90) for PET/CT (Z = 1.58, *P* > 0.05) (Fig. 4). The DOR of DWI and PET/CT was 54.46 (95% CI 17.98–164.99) and 15.77 (95% CI 8.19–30.37), respectively (Fig. 5). The PLR of DWI and PET/CT was 9.58 (95% CI 4.56–20.13) and 4.22 (95% CI 2.87–6.22), while NLR was 0.17 (95% CI 0.11–0.26) and 0.22 (95% CI 0.13–0.38), respectively (Fig. 6).

Regarding the diagnostic accuracy, there was significant heterogeneity for both sensitivity (I^2^ = 83.69 for DWI, I^2^ = 85.58 for PET/CT) and specificity (I^2^ = 81.39 for DWI, I^2^ = 60.75 for PET/CT). While both DWI and PET/CT studies indicated the absence of threshold effect under the Spearman correlation coefficient test [(ρ = − 0.068, *P* = 0.842 for DWI); (ρ = 0.224, *P* = 0.533 for PET/CT)]. Thus, subgroup analyses were independently carried out to assess the potential sources of heterogeneity presented in Tables 3 and 4. It was found that not only lesions included both nodules and masses, but also the sensitivities of DWI and PET/CT increased using histological results as the reference standard. While studies using SUV-CR in PET/CT have higher specificity.

### Publication bias

**Fig. 7:**
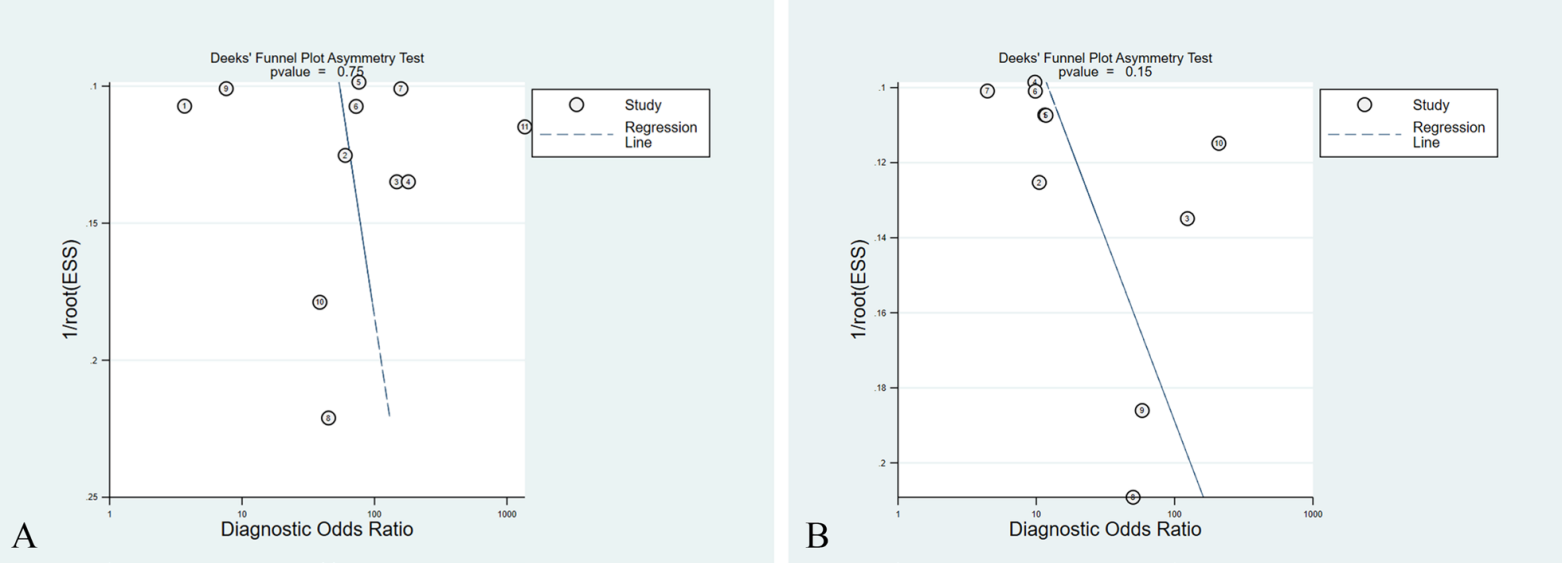
The Deeks’ funnel plot asymmetry test for **A** DWI and **B** PET/CT of 10 eligible studies. One DWI study [24] was counted twice due to the use of both 1.5-T and 3.0-T MRI on all patients, resulting in a total of 11 studies in the table. ESS: effective sample size

In the present meta-analysis, the Deek’s funnel plot asymmetry testing of DWI and PET/CT (*P* = 0.750 and 0.150, respectively) revealed the absence of publication bias (Fig. 7).

## Discussion

Accurate differentiation of malignant tumors from benign tumors is crucial for patient management. Although PET/CT has been used to evaluate suspicious nodules as a recognized technique, it still has some drawbacks, such as high cost and radiation exposure. MRI, as a promising tool, has been recommended for clinical imaging of pulmonary tumors [27], and showed to play an important role in thoracic oncology. In the present meta-analysis, we compared the diagnostic performance of DWI and PET/CT for differentiating the malignant from benign pulmonary nodules and masses.

Spearman correlation coefficient test was performed in our study, and the results showed that there existed an insignificant threshold effect between the logit of sensitivity and the logit of (1-specificity). The current study demonstrated that DWI yielded higher pooled sensitivity (0.85 vs. 0.82), specificity (0.91 vs. 0.81), and AUC (0.94 vs. 0.87, *P* > 0.05) compared with PET/CT. According to a previous review, the diagnostic performance of DWI with different b-values can achieve high sensitivity (70–89%) and specificity (61–97%) [28]. On the other hand, PET/CT derives sensitivity between 49 and 100% as well as specificity between 17 and 85% for this differentiation [18, 21, 29, 30]. Additionally, DOR is a measurement combining sensitivity with specificity to assess diagnostic performance [31]. In our study, the DOR of DWI was higher than that of PET/CT (54.46 vs. 15.77), suggesting that DWI could have a slightly better performance in the differential diagnosis of pulmonary nodules.

As the likelihood ratio has been regarded as a more meaningful estimate in clinical practice, a highly robust diagnostic test might have a PLR > 10 and an NLR < 0.1, while a moderately advantageous one should have a PLR of 5–10 and an NLR of 0.1–0.2[32, 33]. In our study, PLR values of DWI and PET/CT were 9.58 and 4.22, while NLR values of DWI and PET/CT were 0.17 and 0.22, respectively. The PLR of DWI was higher and the NLR was lower than PET/CT, which suggested that DWI could be a moderately advantageous test in clinical practice.

Despite that both imaging modalities have great potential for pulmonary nodule assessment, FP and FN results are inevitable. Some inflammatory diseases, such as fungal infections, have shown FP results on DWI because of the infiltration of inflammatory cells [34]. Meanwhile, Deppen et al. [35] and Croft et al. [36] reported that granulomatous disease could lead to FP scans on PET/CT due to the high glucose metabolism. Besides, some adenocarcinomas, especially well-differentiated type, may represent FN findings on both DWI and PET/CT, because they have lower tumor cellularity and more abundant mucus than other types of carcinoma [34, 37–40]. Usuda et al. [41] reported that after pulmonary resection for lung cancer, DWI is more useful to discriminate suture granuloma from suture recurrence compare with PET/CT. Thus, a study on the mismatched results between PET/CT and DWI should be essentially conducted.

In the present meta-analysis, we included eligible studies published in Chinese to decrease the probability of publication bias. Moreover, we performed subgroup analyses to examine sources of heterogeneity. In the subgroup analyses, lesions that included masses and nodules [22, 24] had higher sensitivities in both DWI (0.87 vs. 0.74) and PET/CT (0.97 vs. 0.77) in comparison with lesions that included only nodules [8, 18, 19, 25]. This may demonstrate that the smaller the lesion size is, the lower the diagnostic accuracy of DWI and PET/CT can be. Khalaf et al. [30] also found that the diagnostic performance of PET/CT depends on lesion diameters; the bigger they are, the higher sensitivity (91–100%) and the lower specificity (17–47%) it has. Furthermore, compared with using histological and follow-up examinations[8, 18, 19, 21, 22, 24, 25] as the reference standard, using only histological examinations[20, 23, 26] would have higher sensitivities in both techniques [DWI (0.82 vs. 0.90); PET/CT (0.75 vs. 0.92)]. It might be because patients with malignant nodules would more likely to undergo surgeries than those with benign nodules; therefore, more malignancies and fewer benign lesions might be included in studies using histology as the reference standard. This could lead to a higher sensitivity in these studies. Moreover, our results showed that SUV-CR [8, 19, 21, 24, 25] derived from PET/CT had a higher specificity compared with the SUV_max_ (0.84 vs. 0.78) [18, 20–23, 26]. In addition, SUV-CR has previously shown a higher sensitivity in comparison with SUV_max_ [8, 42]. SUV-CR, as a semi-quantitative parameter, is not likely affected by factors, such as lesion size, body size, etc., while SUV_max_ could be affected [43]; thus, SUV-CR could be more clinically valuable in discriminating benign nodules from malignant lung nodules.

Our study contains some limitations. First, the risk of bias in all the included studies was high, therefore, high-quality studies should be conducted in the future. Second, most of the studies used follow-up examinations as the reference standard, which might lead to misclassification and potential bias in the result [16, 17]. Third, our meta-analysis did not include unpublished studies, which might ignore some negative or insignificant results.

In conclusion, DWI may have similar performance compared with PET/CT for differentiating the malignant from benign pulmonary nodules or masses. DWI has some advantages over PET/CT in terms of lower cost and no exposure to ionizing radiation. Therefore, DWI could be considered as a potential alternative for differentiating pulmonary lesions. However, prospective studies with higher quality and larger sample sizes should be carried out to validate the clinical value of DWI.

## Supplementary Information

**Additional file 1**. The search strategy of PubMed.

## Data Availability

The data supporting this meta-analysis are from previously reported studies and datasets, which have been cited. The processed data are available from the coresponding author upon request.

## Abbreviations

CT: Computed tomography
PET/CT: Positron emission tomography/computed tomography
MRI: Magnetic resonance imaging
DWI: Diffusion-weighted imaging
ADC: Apparent diffusion coefficient
TP: True-positive
TN: True-negative
FP: False-positive
FN: False-negative
QUADAS-2: Quality Assessment of Diagnostic Accuracy Studies 2
CIs: Confidence intervals
PLR: Positive likelihood ratio
NLR: Negative likelihood ratio
DORs: Diagnostic odds ratios
SROC: Summary receiver operating characteristic
SI-CR: The contrast ratio between the lesions and spinal cord of signal intensity
SUV_max_: The maximum standard uptake value
SUV-CR: The contrast ratio between the lesions and contralateral lung of SUV
AUC: The area under the ROC curve
