# Supplementary material for: Diagnostic performance of diffusion-weighted imaging versus 18F-FDG PET/CT in differentiating pulmonary lesions: an updated meta-analysis of comparative studies
Source: BMC Med Imaging. 2023 Mar 10;23:37. doi: 10.1186/s12880-023-00990-y (PMC10007793; doi:10.1186/s12880-023-00990-y)
Supplement: Supplementary file 1 — Additional file 1. The search strategy of PubMed. [file 12880_2023_990_MOESM1_ESM.docx]

**Search strategy of PubMed**

**((((((((((((((((((((((((((((((((((((((PET-CT Scan[Title/Abstract]) OR (PET-CT Scans[Title/Abstract])) OR (Scan, PET-CT[Title/Abstract])) OR (Scans, PET-CT[Title/Abstract])) OR (PET CT Scan[Title/Abstract])) OR (CT Scan, PET[Title/Abstract])) OR (CT Scans, PET[Title/Abstract])) OR (PET CT Scans[Title/Abstract])) OR (Scan, PET CT[Title/Abstract])) OR (Scans, PET CT[Title/Abstract])) OR (CT PET[Title/Abstract])) OR (PET[Title/Abstract])) OR (Positron Emission[Title/Abstract])) OR (Tomography-Computed Tomography[Title/Abstract])) OR (PET-CT[Title/Abstract])) OR (CT PET Scan[Title/Abstract])) OR (CT PET Scans[Title/Abstract])) OR (PET Scan, CT[Title/Abstract])) OR (PET Scans, CT[Title/Abstract])) OR (Scan, CT PET[Title/Abstract])) OR (Scans, CT PET[Title/Abstract])) OR (Positron Emission Tomography[Title/Abstract])) OR (Positron-Emission Tomography Imaging[Title/Abstract])) OR (Imaging, Positron-Emission Tomography[Title/Abstract])) OR (Positron Emission Tomography Imaging[Title/Abstract])) OR (Positron-Emission Tomography Imagings[Title/Abstract])) OR (Tomography Imaging, Positron-Emission[Title/Abstract])) OR (Tomography, Positron-Emission[Title/Abstract])) OR (Tomography, Positron Emission[Title/Abstract])) OR (PET Scan[Title/Abstract])) OR (PET Scans[Title/Abstract])) OR (Scan, PET[Title/Abstract])) OR (PET Imaging[Title/Abstract])) OR (Imaging, PET[Title/Abstract])) OR (PET Imagings[Title/Abstract])) OR ("Positron-Emission Tomography"[Mesh] OR "Positron Emission Tomography Computed Tomography"[Mesh])) AND (((((((Magnetic Resonance Imaging, Diffusion[Title/Abstract]) OR (Diffusion MRI[Title/Abstract])) OR (Diffusion MRI[Title/Abstract])) OR (Diffusion Weighted MRI[Title/Abstract])) OR (MRI, Diffusion Weighted[Title/Abstract])) OR (DWI[Title/Abstract])) OR ("Diffusion Magnetic Resonance Imaging"[Mesh]))) AND ((((((((((((((((Pulmonary Nodule, Solitary[Title/Abstract]) OR (Nodule, Solitary Pulmonary[Title/Abstract])) OR (Solitary Pulmonary Nodules[Title/Abstract])) OR (Nodules, Solitary Pulmonary[Title/Abstract])) OR (Pulmonary Nodules, Solitary[Title/Abstract])) OR (Pulmonary Coin Lesion[Title/Abstract])) OR (Lesion, Pulmonary Coin[Title/Abstract])) OR (Lesions, Pulmonary Coin[Title/Abstract])) OR (Coin Lesions, Pulmonary[Title/Abstract])) OR (Pulmonary Coin Lesions[Title/Abstract])) OR (Coin Lesion, Pulmonary[Title/Abstract])) OR (Multiple Pulmonary Nodule[Title/Abstract])) OR (Pulmonary Nodule, Multiple[Title/Abstract])) OR (Pulmonary Nodules, Multiple[Title/Abstract])) OR (mass[Title/Abstract])) OR (("Solitary Pulmonary Nodule"[Mesh]) OR "Multiple Pulmonary Nodules"[Mesh]))) AND ((((((((((((((((Lungs[Title/Abstract]) OR (Lung Diseases[Title/Abstract])) OR (Pulmonary Medicine[Title/Abstract])) OR (Pneumonology[Title/Abstract])) OR (Respiratory Medicine[Title/Abstract])) OR (Medicine, Respiratory[Title/Abstract])) OR (Pulmonology[Title/Abstract])) OR (Pneumology[Title/Abstract])) OR (Medicine, Pulmonary[Title/Abstract])) OR (Disease, Lung[Title/Abstract])) OR (Diseases, Lung[Title/Abstract])) OR (Lung Disease Pulmonary Disease[Title/Abstract])) OR (Disease, Pulmonary[Title/Abstract])) OR (Diseases, Pulmonary[Title/Abstract])) OR (Pulmonary Diseases[Title/Abstract])) OR ("Pulmonary Medicine"[Mesh] OR "Lung Diseases"[Mesh] OR "Lung Diseases, Fungal"[Mesh] OR "Lung"[Mesh]))**
